# From Waste to Value: Solubility and Dissolution Enhancement of Bioactive Extracts from Olive Leaves Using Poloxamers

**DOI:** 10.3390/molecules30040928

**Published:** 2025-02-17

**Authors:** Muhammad Wasim, Maria Camilla Bergonzi

**Affiliations:** Department of Chemistry “Ugo Schiff” (DICUS), University of Florence, Via Ugo Schiff 6, 50019 Sesto Fiorentino, Italy; muhammad.wasim@unifi.it

**Keywords:** olive oil waste valorization, circular economy, triterpenes, polyphenols, poloxamer, solid dispersions, solubility, dissolution, permeability

## Abstract

The European Union, producing over 2.5 billion tons of waste annually, has prompted the European Parliament to implement legal measures and encourage the shift towards a circular economy. Millions of tons of biowaste from olive plant leaves are generated annually, resulting in environmental and economic challenges. To address this, the biowaste of olive leaves was valorized, resulting in the extraction of valuable components, triterpenes and polyphenols, which hold potential pharmaceutical, food, or cosmetic applications. Our research involved the formulation of a triterpene extract (TTP70, 70% triterpenes) as a solid dispersion using Poloxamer-188 (P188) and Poloxamer-407 (P407). The solid dispersions were prepared using a kneading method and various extract-to-polymer weight ratios, including 1:1, 1:2, and 1:5. The influence of hydrophilic carriers on the solubility, dissolution profile, and in vitro passive permeability of TTP70 was evaluated. Both carriers and all considered weight ratios significantly improved the solubility of hydrophobic extract and the dissolution of triterpenes. PAMPA experiments demonstrated the efficacy of the formulation in improving the passive permeation of triterpenes. Subsequently, the solid dispersions were physically mixed with a polyphenol-enriched extract (OPA40, 49% of polyphenols) also obtained from olive leaves, and they were used to fill hard gelatin capsules and produce an oral dosage form. The composite formulations improved the dissolution of both classes of constituents.

## 1. Introduction

Biowaste plays a key role in facilitating the transition to a circular economy by not only preventing waste generation but also connecting its potential as a valuable source of valorized product. The concept behind the circular economy is that there is no waste in nature, but each product can be renewed by re-entering a new production cycle. Wide research is currently focused on addressing the challenges associated with transforming biowaste into valuable bioproducts [1]. Olive waste, including byproducts from olive oil production and residuals from tree pruning, represents a significant opportunity for valorization within the circular bioeconomy framework. Approximately 21.4 million tons of waste and byproducts are generated annually in the European Union, comprising olive pomace, wastewater, and pruning residues like leaves and branches [2,3]. Instead of being discarded, these materials can be transformed into valuable bio-based products, bioenergy, and biofertilizers, thereby enhancing resource efficiency and reducing environmental impacts. Effective management of these waste streams not only supports sustainable agricultural practices but also contributes to the economic viability of the olive sector, highlighting the importance of innovative approaches in circular bioeconomy initiatives [4,5,6,7,8].

The olive milling industry generates considerable amounts of olive mill waste (OMW) in a short period, primarily between October and January. Olive mill wastewater averages 0.3 to 1.2 m^3^ per ton of processed olives, accompanied by solid residues of 500 to 735 kg per ton. While OMW is highly polluting due to its acidity and elevated biological oxygen demand, it is also rich in valuable bioactive compounds like anthocyanins and phenolics. These compounds hold potential for various industrial applications, including fertilizers and antioxidants, prompting a shift toward their valorization rather than mere detoxification [9]. Every year, 4.5 million tons of olive leaves are produced worldwide by the olive oil industry, causing a problem, as they must be removed from the fields and mills. Therefore, encouraging the recovery of typically discarded materials, like leaves, presents an innovative approach in the context of a circular and sustainable bioeconomy.

This biomass contains high added value bioactive compounds with high market potential. The leaves are currently used for biomass production or animal feed. Current processing methods of olive leaf waste include thermochemical, biochemical, drying, extraction, and condensation methods. The treated olive leaves are applied in sectors relating to cattle feed, fertilizers, novel materials, energy generation, and food and pharmaceutical products [10].

However, many high-value-added compounds obtained from olive leaves (polyphenols, triterpenoids, essential oils, lipids, lignocellulose) have high potential, but their practical application is limited by issues such as poor water solubility, permeability, and bioavailability [11]. *Olea europaea* L., commonly known as the olive tree, is valued for its significant contribution to olive oil production, which is highly valued in human nutrition. Moreover, this plant is rich in bioactive secondary metabolites from various chemical classes found in its various parts such as the bark, roots, wood, and leaves [12]. Olive leaves, which have been extensively used in traditional medicine in the Mediterranean area, are rich in polyphenols and triterpenes, and they can be used as a cheap source of high-added-value compounds.

This study is part of the European Union’s Horizon 2020 project aimed at valorizing waste from olive oil production, in particular olive leaves, to make products for food, feed, health, cosmetics, and the pharmaceutical and chemical industries. A set of green extraction technologies was developed and applied for the cascade extraction and fractionation of olive leaf bioactive compounds, and different extracts with different percentages of phytoconstituents or single compounds were obtained. The produced fractions contained (i) polyphenols, (ii) triterpenic acids, (iii) non-polar fraction (essential oils, lipids), (iv) fermented leaf-based products, and (v) lignocellulose-based products. Novel green technologies like enzymatic-assisted extraction and surfactant-assisted extraction, in combination with traditional methods like solid–liquid percolation, pressurized liquid extraction, ultrasound liquid extraction, as well as supercritical fluid extraction, enzymatic hydrolysis, and lignin depolymerization, have been employed to extract the main bioactive compounds (https://oleaf4value.eu/, accessed on 15 February 2025).

In this study, two extracts obtained from olive leaves were combined: a pentacyclic triterpene-enriched extract (TTP70) and a polyphenolic-enriched extract (OPA40) [13]. The fruits and leaves of *Olea europaea* L. contain various triterpenoids, and the triterpene content in the leaves includes high amounts of oleanolic acid (3.0–3.5% dry weight), a significant concentration of maslinic acid (0.50–0.75% dry weight), and minor amounts of ursolic acid (0.20–0.25% dry weight), erythrodiol, and uvaol, which are present in comparable amounts, in a range of 0.05–0.15% dry weight [14]. Triterpenes are known to have multiple benefits for human health [13,15,16]. The literature extensively explores various pharmacological activities, including anti-inflammatory, antioxidant, anti-hyperglycemic, anti-hyperlipidemic, anti-glycation, cardioprotective, hepatoprotective, and chemoprotective effects, as well as effects on epidermal cells and SARS-CoV-2. However, their poor water solubility, permeability, and bioavailability present limitations to their therapeutic application.

Furthermore, in this research, an OPA40 with high content of total polyphenols (49% *w*/*w*), with 41% *w*/*w* corresponding to oleuropein, was used [13]. Polyphenols have been widely described in the literature for their multiple bioactive properties related to their antioxidant and free radical scavenger activities [17]. Many of their pharmacological activities have been attributed to their potent antioxidant efficacy, such as hypoglycemic, antiviral, antimicrobial, platelet-antiaggregant, hypolipidemic, anti-inflammatory, antifungal, and anti-tumoral activities, although their ability to treat oxidant- and inflammatory-related diseases has also been demonstrated (i.e., cancer, cardiovascular disease, hepatic disorder, obesity, diabetes, etc.) [14,18].

Since both of the studied extracts have shown compromised therapeutic applications, new drug delivery systems have been developed to improve their bioavailability, permeability, solubility, and activity [19,20,21,22], such as microemulsions and solid dispersions in the case of oleanolic acid [11,19]. Solid dispersion (SD) refers to the dispersion of one or more compounds in an inert hydrophilic carrier matrix in the solid state, which can either be a small molecule or a polymer [23,24,25]. It often involves a molecular mixture of a drug and a hydrophilic polymer that enhances the drug’s solubility and bioavailability, where the dispersed compounds may exist as individual molecules or clusters, such as particles [23,24,25]. These systems may include various forms such as eutectic mixtures, crystalline solutions, amorphous solid dispersions, or crystalline and amorphous suspensions [23,24,25]. Compared with other strategies for improving the solubility, dissolution rate, and bioavailability of poorly water soluble drugs, SDs are easier to produce and provide a more applicable approach, since they are more effective in particle size reduction with consequent improvements in drug release [24,25]. They enhance particle wettability and porosity; they allow the drugs to be in an amorphous state—which does not require energy to break up the crystal lattice during the dissolution process—and they are found to be more therapeutically compliant by patients since they are solid oral dosage forms [24].

This study aims to use the SD method as an effective approach to enhance the solubility, dissolution profile, and in vitro passive permeability of the TTP70 extract. Subsequently, the SDs were combined in different ratios with OPA40, and the solubility and dissolution profile of the resulting composite formulations were evaluated. By improving these key parameters, this research intends to make the extracts more suitable for pharmaceutical, food, or cosmetic applications, thereby promoting the efficient valorization of olive leaf bio-waste from olive oil production.

## 2. Results

### 2.1. Solubility Studies of TTP70-SDs

We employed the solid dispersion approach to deliver TTP70, a triterpene-enriched extract obtained from olive leaves. As previously reported [13], TTP70 contains 65.34 ± 1.06% *w*/*w* of triterpenes (TTP), such as maslinic acid, oleanolic acid, ursolic acid, uvaol, and erythrodiol. The qualitative and quantitative composition of TTP70, along with its chromatogram at 210 nm, is reported in Supplementary Material Table S1 and Figure S1, respectively. These compounds are insoluble in water, and to improve their aqueous solubility, the TTP70-SDs were developed using P188 and P407 as carriers [19].

In this study, TTP70:poloxamer weight ratios of 1:1, 1:2, and 1:5 were tested, using kneading (K) as the preparation method and physical mixtures (PMs) for comparison. Both hydrophilic polymers significantly enhanced the solubility of TTP in the extract already in the PM, particularly at weight ratios of 1:2 and 1:5 (Table 1, Table 2 and Table 3). The solubility of TTP in a 1:1 TTP70/poloxamer weight ratio was 83 and 85 μg/mL for PM and 117 and 162 μg/mL for P188 and P497 solid dispersions, respectively. The solubility increased to 483 µg/mL and 359 µg/mL for P188 and P407, respectively, at a 1:2 weight ratio. In the case of an extract-to-polymer ratio of 1:5, the TTP solubility further improved compared to the 1:1 ratio, though the enhancement was less pronounced than that observed for the 1:2 ratio, 216 μg/mL for P188 and 268 μg/mL for P407. Based on these promising results, the 1:2 and 1:5 weight ratios were selected for further investigation.

### 2.2. Dissolution Study of TTP70-SDs

**Figure 1:**
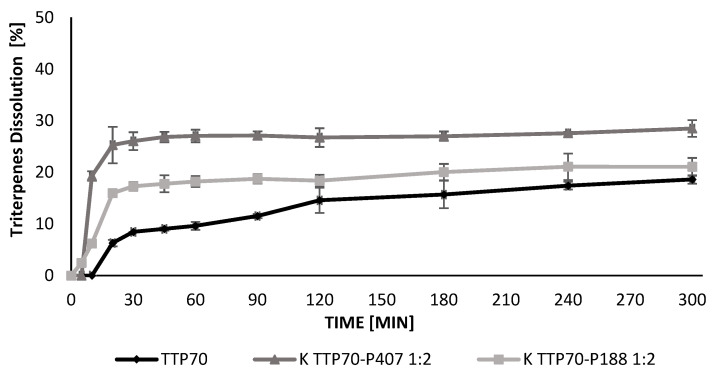
Triterpenes dissolution profiles from extract, kneading solid dispersion extract/P407 1:2, and kneading solid dispersion extract/P188 1:2. Data are expressed as mean ± SD of n = 3 experiments.

**Figure 2:**
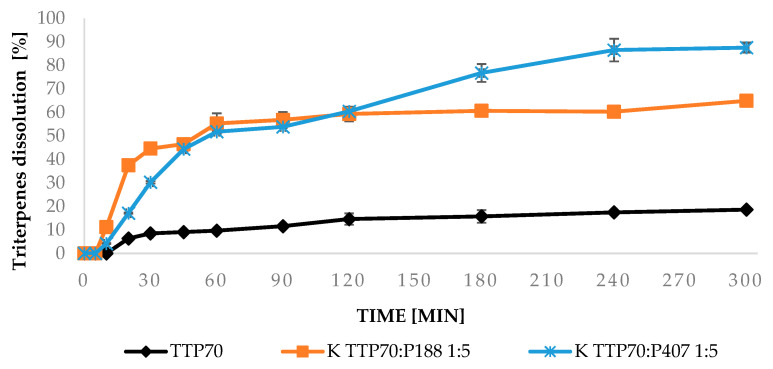
Triterpene dissolution profiles from extract, kneading solid dispersion extract/P407 1:5 and kneading solid dispersion extract/P188 1:5. Data are expressed as mean ± SD of n = 3 experiments.

The dissolution–time profiles of TTP released from TTP70-SDs prepared using the K method were evaluated for P407 and P188 at TTP70/polymer weight ratios of 1:2 and 1:5 compared with TTP70 alone (Figure 1 and Figure 2). In the case of the extract, TTP was gradually released, reaching approximately 18% after 5 h. In contrast, the dissolution of TTP reached 22% with P188 and 28% with P407 for the 1:2 weight ratio, increasing significantly to 65% and 86%, respectively, for the 1:5 ratio.

### 2.3. PAMPA Studies

This test evaluated the effects of the solid dispersion with poloxamers (TTP70-SDs 1:5) on the passive permeability of active constituents. No permeation was observed for TTP from a solution after 6 h. Instead, both SDs improved the passive permeation, with similar effects for both poloxamers. The P_app_ was 4.90 × 10^−5^ ± 6.87 × 10^−6^ cm/s for P407 SD and 6.46 × 10^−5^ ± 6.56 × 10^−6^ cm/s for P188 SD after 2 h, with a recovery greater than 95% [26]. This means there was neither significant TTP membrane retention nor compound binding to plastic surfaces.

### 2.4. Composite Formulations: Solubility Study

To valorize the wastes of olive oil production, in this study, a formulation containing both the triterpene extract TTP70 and the polyphenolic extract OPA40 obtained from olive leaves was prepared. The qualitative and quantitative composition of OPA40, along with the chromatograms, is reported in Supplementary Material Table S2 and Figure S2, respectively. The TTP70-SDs were mixed in a 1:1 ratio with OPA40, referred to as TTP and polyphenol (PP) content. The solubility of PP and TTP depends on the polymer itself and the extract-to-polymer weight ratio (Table 4). Polyphenols have good aqueous solubility; in fact, oleuropein, the main constituent of OPA40, has an aqueous solubility of about 12 mg/mL and reaches a value of approximately 47 mg/mL in the composite formulations. The solubility of triterpenes increased up to 1 mg/mL.

### 2.5. Composite Formulations: Dissolution Study

**Figure 3:**
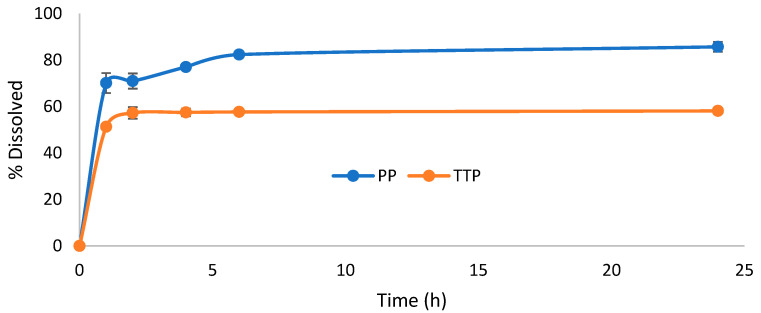
Dissolution profile of triterpenes and polyphenols from 1:1 composite formulation containing solid dispersion TTP70-P188 1:5 and polyphenolic extract OPA40. Data are expressed as mean ± SD of n = 3 experiments.

**Figure 4:**
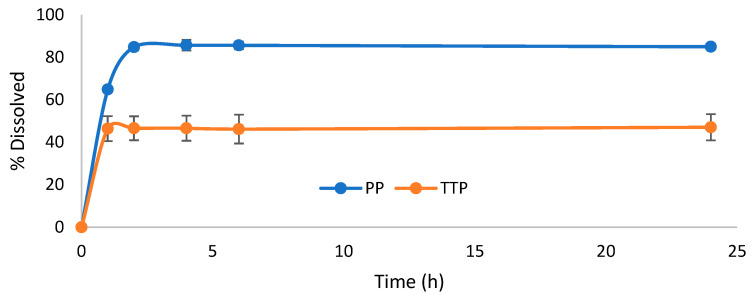
Dissolution profile of triterpenes and polyphenols from 1:1 composite formulation containing solid dispersion TTP70-P407 1:5 and polyphenolic extract OPA40. Data are expressed as mean ± SD of n = 3 experiments.

**Figure 5:**
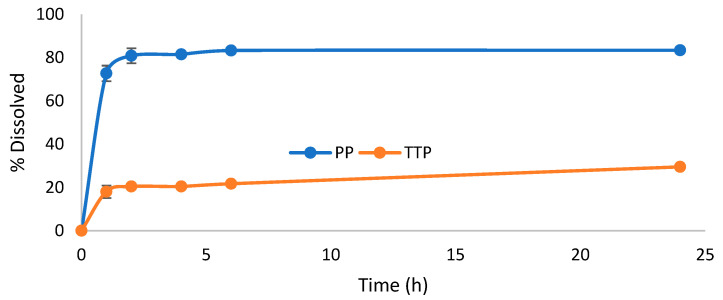
Dissolution profile of triterpenes and polyphenols from 1:1 physical mixture containing triterpene extract and polyphenolic extract. Data are expressed as mean ± SD of n = 3 experiments.

Based on the solubility results, composite formulations of TTP70/poloxamer at a 1:5 SD ratio were mixed in a 1:1 ratio with OPA40 extract. Hard gelatin capsules were prepared by filling them with this solid mixture to simulate an oral dosage form. The dissolution profiles of TTP and PP were evaluated over a 24 h period using a paddle dissolver. An aqueous solution containing 0.3% *w*/*v* SDS was selected as the dissolution medium (Figure 3 and Figure 4). The results were compared to a formulation containing TTP70/OPA40 in a 1:1 ratio (Figure 5).

The PP dissolution reached approximately 70% in 1 h, increasing to 80% in 2–4 h, then it remained constant. The same profile was observed for the case of OPA40 alone, where the final percentage of PP dissolved reached 68% (Figure S3, Supplementary Material). In contrast, TTP was released gradually from the TTP70 over the first 2 h in the samples without poloxamers, stabilizing at around 29% after 24 h (Figure 5). The same dissolution profile was observed in the case of free TTP70 extract (Figure S4, Supplementary Material).

Considering the TTP70-P407 SD/OPA40 composite formulation, TTP dissolution reached 46% in 2 h, and it remained constant through 24 h. Similarly, the P188 SD formulation demonstrated a final percentage of TTP dissolved of 58% in the same period. A gradual dissolution of PP from OPA40 was observed in the composite formulations, with a 10% increase in dissolved amount.

## 3. Discussion

This study is part of the European Union’s Horizon 2020 project aimed at valorizing waste from olive oil production to make products of pharmaceutical, cosmetic, and food interest. The use of herbal extracts has increased worldwide due to their nutritional, cosmetic, and therapeutic properties. Despite outstanding results in experimental in vitro models, herbal extracts often show a lower or negligible in vivo activity due to their poor solubility, resulting in poor absorption and hence poor bioavailability. The extract is a complex matrix that is easier to obtain from the herbal drug than single compounds and is therefore easier to commercialize. Furthermore, using the extract without isolating the components might be beneficial due to the synergistic effects of the constituents.

However, formulating an extract is more difficult than formulating a single compound because the former contains substances with different chemical characteristics and also unidentified compounds. These can contribute to the activity and influence the properties of the phytocomplex. In a previous study [19], the solid dispersion technique was successfully applied to oleanolic acid, yielding excellent results in terms of improved solubility, dissolution, and permeability. A wide range of polymers, including poloxamers, Soluplus, PEGs, and cyclodextrins, were screened using various preparation methods. The present study builds upon the previous findings, applying the same approach to deliver TTP70 extract. Triterpenes have an aqueous solubility of approximately 7 µg/mL. Solid dispersions were prepared using P188 and P407 as hydrophilic carriers. In a previous study, both carriers demonstrated excellent performance in improving the solubility and permeability of oleanolic acid [19]. Poloxamers were selected because they are GRAS (generally recognized as safe) excipients, and they have been widely used in the development of many pharmaceutical formulations and proposed for various applications ranging from the targeting of the central nervous system to drug delivery, gene therapy, tissue engineering, and diagnostics [27,28,29]. Poloxamer 407 is an excipient of various formulations that is approved by the U.S. Food and Drug Administration (FDA) for pharmaceutical application [30].

All the weight ratios tested in this study produced a strong increase in the solubility of the triterpenes already evident in the PM. The SDs produced a further increase, with the 1:2 and 1:5 ratios being the best. P188 and P407 are amphiphilic poloxamers and they form micellar aggregates in an aqueous medium, promoting the solubilization of the hydrophobic triterpenes. In the case of the extract-to-polymer ratio of 1:5, the TTP solubility further improved compared to the 1:1 ratio, though the enhancement was less pronounced than with the 1:2 ratio.

Furthermore, both poloxamers substantially enhanced the dissolution profile of the extract, with greater effects observed at higher extract-to-polymer ratios. P407 demonstrated superior performance between the two poloxamers, consistent with findings for oleanolic acid [19]. Poloxamers are triblock copolymers, where the central hydrophobic block is a polypropylene glycol structure, and the external portions are two hydrophilic blocks of polyethylene glycol (PEG). In P407, the approximate lengths of the two PEG portions are 101 units, while the approximate length of the propylene glycol block is 56 repeat units, giving it an HLB of 18–23. The propylene oxide groups in P188 are about 29 units, and the polyethylene oxide groups are about 152 PEG, with an HLB value > 24. Due to its relatively more lipophilic nature, P407 is better able to solubilize the lipophilic components of the extract.

The effect of SDs on the passive permeability of TTP was evaluated with the PAMPA assay across an artificial membrane that mimics the intestinal barrier. TTP are compounds that do not permeate, but both poloxamers improved their passive permeability. These findings demonstrated the efficacy of the formulation in improving not only the solubility and dissolution of the extract but also its passive permeation, as already demonstrated for oleanolic acid [19].

During the production of olive oil, large amounts of waste and byproducts are produced. These products are a great source of high-added-value compounds, which can be used as food additives and/or nutraceuticals in the food, cosmetic, and pharmaceutical industries. SDs can constitute a promising tool to recover high-added-value compounds from olive oil wastes and byproducts and to improve the biopharmaceutical properties of phytoconstituents. In order to valorize the wastes of olive oil production, in this study, a formulation containing both the triterpene extract TTP70 and a polyphenolic extract OPA40 obtained from olive leaves was prepared. The TTP70-SDs were mixed in a 1:1 ratio with OPA40, referred to as the TTP and PP content.

The solubility of polyphenols and triterpenes depends on the polymer itself and the extract-to-polymer weight ratio (Table 4). However, for all the composite formulations, a high increase in the solubility was observed. The solubility of oleuropein, the main constituent of OPA40, was found to be increased by approximately four-fold in the composite formulations. The solubility of TTP increased by four orders of magnitude, reaching values higher than those obtained with the SDs. This may be due to a solubilizing effect exerted by the constituents of the two extracts, both known and undetermined, which can influence the solubilization process of the lipophilic constituents in addition to that produced by the hydrophilic carriers.

The composite formulations improved the PP dissolution of about 10% with respect to free OPA40 and the TTP dissolution of 15–20% with respect to the 1:1 physical mixture of the two extracts. These results align with the dissolution profiles observed in the earlier test of TTP70 SDs (Figure 2), confirming the effectiveness of the formulations in enhancing TTP dissolution.

A gradual dissolution of PP from OPA40 was observed in the composite formulations, with a 10% increase in the dissolved amount. This result highlights the positive synergistic effect of the TTP70 phytocomplex and poloxamers on PP dissolution. Additionally, a prolonged and enhanced dissolution profile for TTP70 was achieved. This consistency not only can represent an important aspect to enhance the bioavailability of the active compounds but also highlights the effectiveness of solid dispersions in pharmaceutical, cosmetic, or food applications. Moreover, this approach facilitates the development of more efficient delivery systems while contributing to the valorization of agricultural biowaste, particularly olive leaf extracts.

## 4. Materials and Methods

### 4.1. Materials

Natac Biotech SL (Getafe, Madrid, Spain) provided the two *Olea europaea* L. leaf extracts: triterpenes-enriched extract (TTP70) and polyphenols extract (OPA40). The preparation of the extracts has been previously reported [13]. Poloxamers, sodium dodecyl sulphate, ethanol, and HPLC-grade solvents were provided by Sigma Aldrich Italia (Milan, Italy). PAMPA was from Millipore Corporation (Tullagreen, Carrigtwohill, County Cork, Ireland).

### 4.2. Preparation of SDs

Firstly, a physical mixture (PM) of TTP70 and the selected polymers was prepared by mixing ground extract and polymer in a mortar. The components were mixed at extract-to-polymer weight ratios of 1:1, 1:2, and 1:5. The hydrophilic polymers selected for solid dispersion preparation were the ones that showed better improvements in solubility of oleanolic acid in a previous study [19]: Poloxamer 407 (P407) and Poloxamer 188 (P188). For the preparation of SDs with the kneading method, the PM was mixed with ethanol enough to maintain a slightly moist consistency. After 10 min of kneading in the mortar, the solid was placed in a glass desiccator overnight to ensure the removal of any residual moisture and ethanol [19].

### 4.3. Chromatography Conditions

For the chromatographic analysis, an HP1100 liquid chromatograph coupled with DAD detector was used (Agilent Technologies, Palo Alto, CA, USA). For quality and quantitative analysis of TTP70 extract, a Luna Omega Polar C18 (150 × 4.6 mm, 3 µm, Agilent Technology, Santa Clara, CA, USA) was used as an analytical column. The analytical method consisted of (A) CH_3_CN and (B) H_2_O pH 3.2 (by HCOOH) as mobile phases, applying isocratic conditions: 85% A and 15% B for 25 min. The flow rate was 0.5 mL/min. The acquisition wavelength was 210 nm. A stock solution of TTP70 in CH_3_OH (0.652 mg/mL) was prepared for the calibration curve. From this solution, five different dilutions were made. The R^2^ value was 0.9999.

For quality and quantitative analysis of OPA40, the compounds were detected at 233 nm with an eluent flow rate of 0.4 mL/min, with (A) acetonitrile and (B) water pH 3.2 (by formic acid) as mobile phases, and with a gradient analytical method starting at 5% A and 95% B, 40% A and 60% B at 30 and 35 min, 100% A and 0% B at 40 and 43 min, and 5% A and 95% B at 50 min. For the calibration curve, a standard stock solution of OPA40 in methanol (0.475 mg/mL) was prepared. Five different dilutions were made from the mother solution. The calculated R^2^ value was 0.9999.

### 4.4. Characterization of SDs

#### 4.4.1. Solubility Studies

To determine the solubility of TTP70, TTP70-SDs, and the physical mixture of OPA40/TTP70-SDs, an excess amount of sample was added to 2 mL of deionized water (Simplicity UV Water Purification System, Merck Millipore, Darmstadt, Germany) and maintained under stirring at room temperature for 24 h. The dispersion was then centrifuged at 14,000 rpm for 15 min, and the supernatant was analyzed using HPLC-DAD analysis. All solubility experiments were performed in triplicate.

#### 4.4.2. In Vitro Dissolution Studies

In vitro dissolution studies were performed to evaluate the extent of dissolution of TTP70, TTP70-SDs, and the physical mixture of OPA40/TTP70-SDs. The test was carried out according to the European Pharmacopoeia using a paddle dissolver (AT7 Dissolver, equipped with basket apparatus, Apparatus 1 E.P., Sotax, Basel, Switzerland) [31]. Type 1 gelatin capsules were selected and filled with pure TTP70, TTP70-SDs, or composite formulations, each containing a TTP70 amount corresponding to 20 mg of triterpenes (TTP). The capsules were immersed in 900 mL of an aqueous solution containing SDS 0.3% *w*/*v* at 37 °C, and the paddle was set at 100 rpm. At predetermined time intervals, 2 mL of dissolution medium was withdrawn, and to guarantee sink conditions, 2 mL of fresh medium was added. All samples were centrifuged at 14,000 rpm for 15 min, and the final concentration of triterpenes and polyphenols was determined using HPLC-DAD. The experiments were performed in triplicate.

#### 4.4.3. Parallel Artificial Membrane Permeability Assay (PAMPA)

PAMPA was used to evaluate the permeation of TTP70 from the donor compartment to the acceptor compartment. An EtOH:PBS (95:5) solution was used as an acceptor medium, and the membranes of the filter plates were covered with lecithin and cholesterol in 1,7-octadiene. A TTP70 or TTP70-SDs solution was inserted into the donor chamber. The system was incubated at 25 °C for 1 and 2 h. Then, the samples were centrifuged at 14,000 rpm and diluted with MeOH before the HPLC-DAD analysis. The permeability coefficient (*P_e_*, cm/s) was calculated as follows:

$$P_{e}=\frac{-\ln\left[1-\frac{C_{At}}{C_{eq}}\right]}{A\left(\frac{1}{V_{D}}+\frac{1}{V_{A}}\right)t}$$

Here, *A* represents the active surface area (0.3 cm^2^ × the apparent porosity of the filter), *V_D_* and *V_A_* denote the well volumes of the donor and acceptor plates (in mL), respectively, *t* is the incubation time (in seconds), and *C_At_* and *C_Dt_* represent the concentrations of TTP70 in the acceptor and donor plates at time *t*, respectively. The equilibrium concentration (*C_eq_*) was calculated using the following equation:

$$C_{eq}=\frac{\left[C_{Dt}\times V_{D}+C_{At}\times V_{A}\right]}{V_{A}+V_{D}}$$

The experiments were performed in quadruplicate.

#### 4.4.4. Preparation of Composite Formulations

OPA40 and TTP70-SDs were mixed in a mortar in weight ratios of 1:1, 5:1, and 1:5 based on the percentage of active constituents in the extracts.

### 4.5. Statistical Analysis

The experiments were repeated three times, and the results were expressed as means ± standard deviations. Statistical significance was calculated using Student’s *t*-test. Differences were considered significant for * *p* < 0.05, ** *p* < 0.01, *** *p* < 0.001. Graph Pad Prism (Graph Pad Software 8, San Diego, CA, USA) was used for performing all statistical analysis.

## 5. Conclusions

Solid dispersions constitute a promising tool to recover high-added-value compounds from olive oil waste and byproducts and to improve the biopharmaceutical properties of their phytoconstituents. This research aimed to develop a preparation containing both a triterpene extract formulated as a solid dispersion and a polyphenolic extract. The poloxamers used in the preparation of solid dispersions of TTP70 enhanced the solubility of the triterpenes, as well as their dissolution profile and in vitro permeation. The solubility and dissolution were further increased in composite formulations by the presence of OPA40. The developed formulation allows us to simultaneously deliver triterpenes and polyphenols extracted from olive leaves, obtaining a product that maintains the pharmacological and nutraceutical properties of both classes of compounds with a good solubility and dissolution profile. Furthermore, the optimized composite formulations can be easily marketed by transforming them into capsules or tablets.

## Figures and Tables

**Table 1:** Solubility of TTP in 1:1 TTP70/poloxamer weight ratio. PM: physical mixture; K: kneading. Data are expressed as mean ± SD of n = 3 experiments. ** *p* < 0.01. NS: not statistically significant.

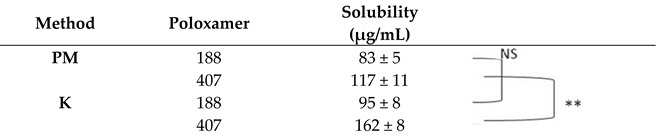

**Table 2:** Solubility of TTP in 1:2 TTP70/poloxamer weight ratio. PM: physical mixture; K: kneading. Data are expressed as mean ± SD of n = 3 experiments. *** *p* < 0.001.

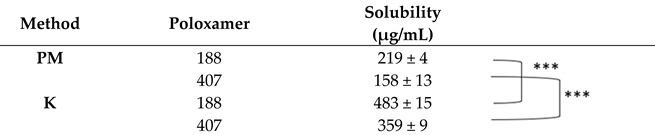

**Table 3:** Solubility of TTP in 1:5 TTP70/poloxamer weight ratio. PM: physical mixture; K: kneading. Data are expressed as mean ± SD of n = 3 experiments. ** *p* < 0.01. NS: not statistically significant.

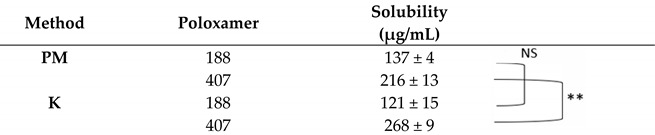

**Table 4:** Solubility of PP and TTP in the 1:1 physical mixture obtained with TTP70:P188 SD or TTP70:P407 SD (1:1, 1:2, or 1:5 weight ratio) and OPA40. Data are expressed as mean ± SD of n = 3 experiments. PP = polyphenols; TTP = triterpenes.

Polymer	SD TTP70:Poloxamer	PP Solubility (mg/mL)	TTP Solubility (mg/mL)
P407	1:1	43.72 ± 1.07	0.45 ± 0.06
P188		46.48 ± 1.55	0.22 ± 0.04
P407	1:2	37.87 ± 2.69	0.77 ± 0.07
P188		38.29 ± 3.15	0.39 ± 0.03
P407	1:5	42.11 ± 1.95	0.96 ± 0.09
P188		46.67 ± 2.60	0.68 ± 0.05

## Data Availability

The data are contained within this article.

## Supplementary Materials

The following supporting information can be downloaded at: https://www.mdpi.com/article/10.3390/molecules30040928/s1, Table S1: Qualitative–quantitative composition of TTP70 (%*w*/*w*), Table S2: Qualitative–quantitative composition of OPA40 (%*w*/*w*), Figure S1: Chromatographic profile of TTP70. Maslinic acid: 4.59 min, oleanolic acid: 8.56 min, ursolic acid: 8.72 min, uvaol: 12.16 min, erythrodiol 12.55 min, Figure S2: Chromatographic profiles of OPA40, Figure S3: Dissolution profile of polyphenols (PP) from OPA40, Figure S4: Dissolution profile of triterpenes (TTP) from TTP70.
