# Supplementary material for: From Waste to Value: Solubility and Dissolution Enhancement of Bioactive Extracts from Olive Leaves Using Poloxamers
Source: Molecules. 2025 Feb 17;30(4):928. doi: 10.3390/molecules30040928 (PMC11858259; doi:10.3390/molecules30040928)

Article

From Waste to Value: Solubility and Dissolution Enhancement of Bioactive Extracts from Olive Leaves Using Poloxamers

Muhammad Wasim and Maria Camilla Bergonzi *

Department of Chemistry "Ugo Schiff" (DICUS), University of Florence, Via Ugo Schiff 6, 50019 Sesto Fiorentino, Italy; muhammad.wasim@unifi.it
* Correspondence: mc.bergonzi@unifi.it

Table S1. Quali-quantitative composition of TTP70 (%*w*/*w*).

Compound	%*w*/*w*
Oleanolic acid	36.73
Maslinic acid	14.41
Uesolic acid	8.63
Erythrodiol	0.97
Uvaol	4.61
Total	65.34

Table S2. Quali-quantitative composition of OPA40 (%*w*/*w*).

Compound	OPA40
Oleuropein	41.67
Hydroxytyrosol	0.16
Verbascoside	1.18
Oleuroside	4.39
Luteolin	0.02
Luteolin-7-O-glucoside	1.05
Luteolin-4-O-glucoside	0.46
Luteolin diglucoside	0.06
Apigenin-7-O-glucoside	0.17
Apigenin-7-O-rutinoside	0.13
7-epilogalin	0.30
Elenolic acid glucoside	0.04
Total	49.63

Figure S1. Chromatographic profile of TTP70. Maslinic acid: 4.59 min, oleanolic acid: 8.56 min, ursolic acid: 8.72 min, uvaol: 12.16 min, erythrodiol 12.55 min.

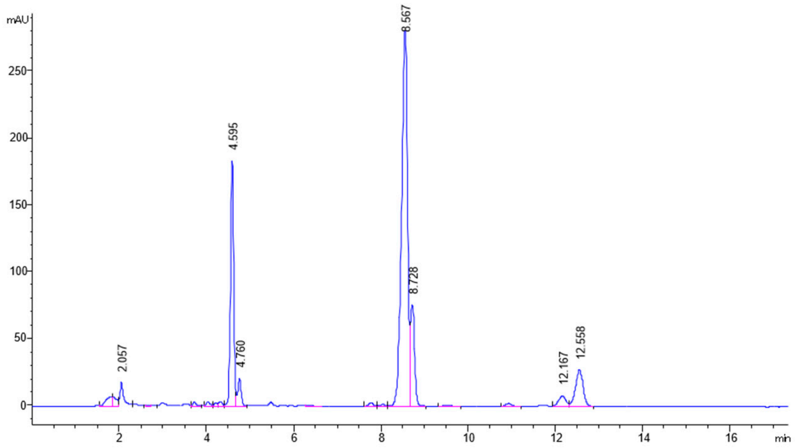

Figure S2. Chromatographic profiles of OPA40.

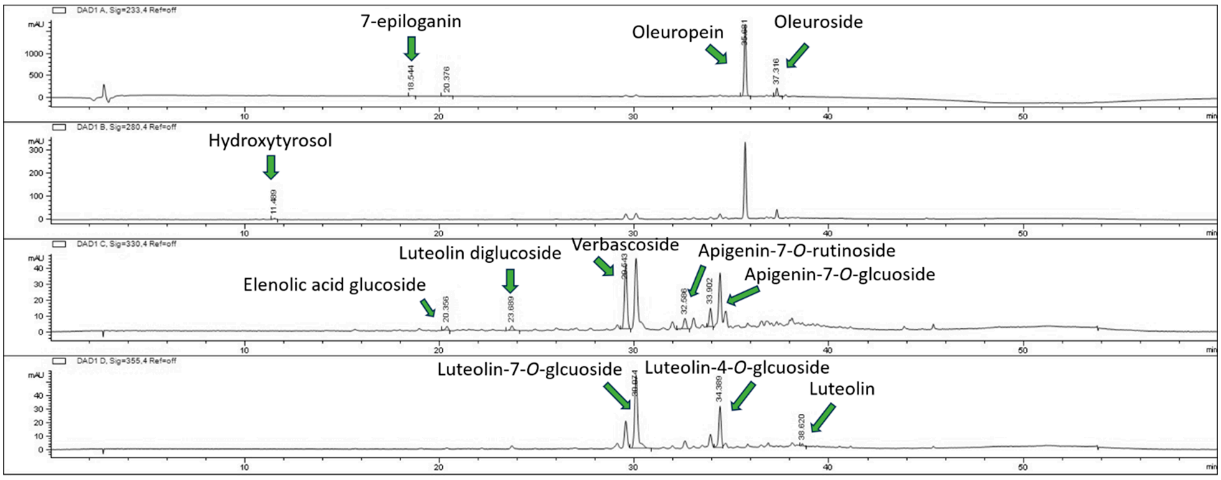

Figure S3. Dissolution profile of polyphenols (PP) from OPA40. Data are expressed as mean ± SD of n=3 experiments.

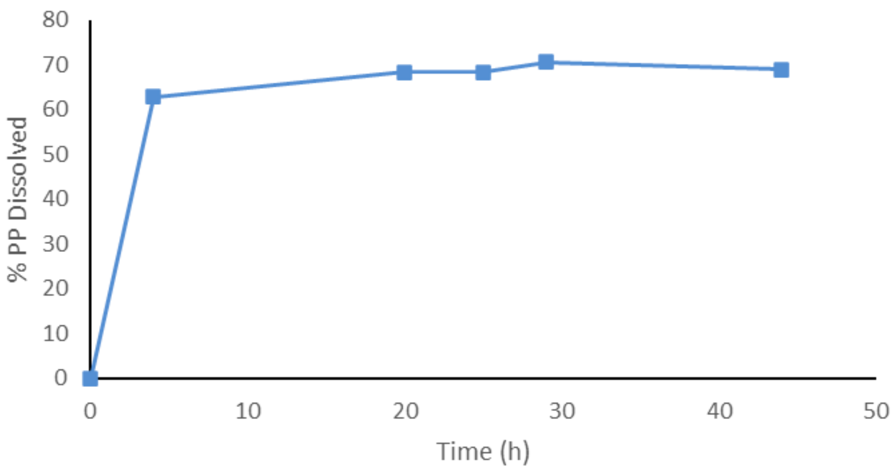

Figure S4. Dissolution profile of triterpenes (TTP) from TTP70. Data are expressed as mean ± SD of n=3 experiments.

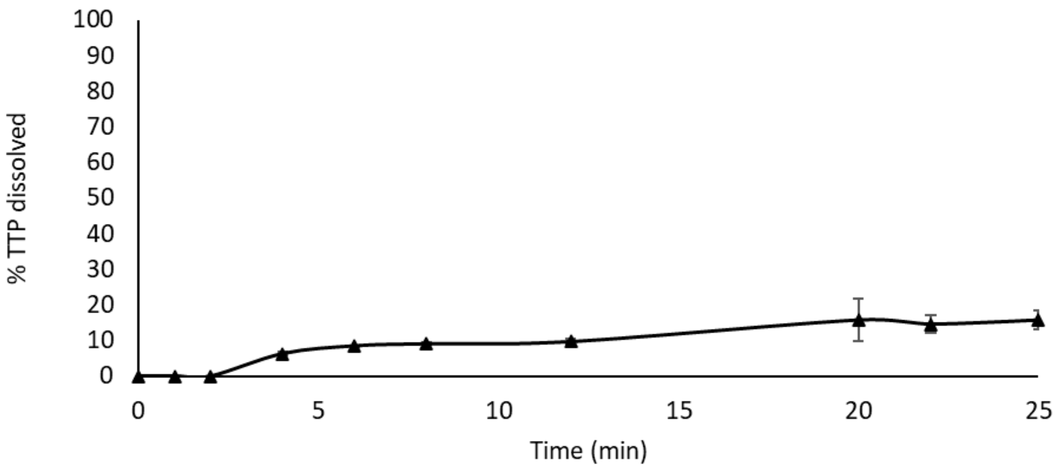

Supplement: Supplementary file 1 [file molecules-30-00928-s001.zip › molecules-3450225-supplementary.pdf]
